# No evidence for an attentional bias towards implicit temporal regularities

**DOI:** 10.3758/s13414-019-01851-z

**Published:** 2019-09-04

**Authors:** Atser Damsma, Niels Taatgen, Ritske de Jong, Hedderik van Rijn

**Affiliations:** 1 Department of Psychology, University of Groningen, Grote Kruisstraat 2/1, 9712 TS Groningen, the Netherlands; 2 Bernoulli Institute, University of Groningen, Groningen, the Netherlands

**Keywords:** Attentional bias, Temporal regularity, Statistical learning, Visual search

## Abstract

Action and perception are optimized by exploiting temporal regularities, and it has been suggested that the attentional system prioritizes information that contains some form of structure. Indeed, Zhao, Al-Aidroos, and Turk-Browne (*Psychological Science, 24*(5), 667–677, 2013) found that attention was biased towards the location and low-level visual features of shapes that appeared with a regular order but were irrelevant for the main search task. Here, we investigate whether this bias also holds for irrelevant metrical temporal regularities. In six experiments, participants were asked to perform search tasks. In Experiments 1a–d, sequences of squares, each presented at one of four locations, appeared in between the search trials. Crucially, in one location, the square appeared with a regular rhythm, whereas the timing in the other locations was random. In Experiments 2a and 2b, a sequence of centrally presented colored circles was shown in between the search trials, of which one specific color appeared regularly. We expected that, if attention is automatically biased towards these temporal regularities, reaction times would be faster if the target matches the location (Experiments 1a–d) or color (Experiments 2a–b) of the regular stimulus. However, no reaction time benefit was observed for these targets, suggesting that there was no attentional bias towards the regularity. In addition, we found no evidence for attentional entrainment to the rhythmic stimulus. These results suggest that people do not use implicit rhythmic temporal regularities to guide their attention in the same way as they use order regularities.

When interacting with the environment, humans extract and exploit regularities in order to make inferences or anticipate future events. This kind of statistical learning can be used to optimize perception, motor timing, and the allocation of attentional resources. Indeed, sensitivity to statistical regularities has been found to occur over a wide range of stimuli and different modalities, such as in regular spatial arrangements (Biederman, Mezzanotte, & Rabinowitz, 1982; Chun & Jiang, 1998; Fiser & Aslin, 2001), implicit artificial grammar (Reber, 1967; Saffran, Aslin, & Newport, 1996), and the order of presented shapes (Turk-Browne, Scholl, Chun, & Johnson, 2009). Interestingly, statistical learning often occurs without explicit knowledge or instructions about the regularities, indicating that it is an automatic and implicit process (e.g., Turk-Browne, Jungé, & Scholl, 2005).

In line with these examples, humans pick up temporal regularities in their environment rather automatically (Damsma & van Rijn, 2017; Large & Palmer, 2002; Povel, 1981). Temporal regularities can be exploited to predict the timing of upcoming events and thereby allow one to prepare an efficient response (Nobre & van Ede, 2018). For example, reaction times decrease when a target stimulus appears predictably after a specific foreperiod (Niemi & Näätänen, 1981). In addition, attention can be directed by temporal structures in a similar way as by predictable spatial arrangements, leading to temporal contextual cuing (Olson & Chun, 2001). That is, Olson and Chun (2001) found that predictable sequences of event durations preceding a target lead to faster reaction times.

In a similar way, humans are sensitive to rhythmic events in their environment. When a stimulus occurs with an isochronous rhythm, attention can be synchronized to the stimulus through entrainment (Lakatos, Karmos, Mehta, Ulbert, & Schroeder, 2008; Large & Jones, 1999). In this way, neural entrainment to rhythmic stimuli has been shown to facilitate perception, such as in pitch judgment (Jones, Moynihan, MacKenzie, & Puente, 2002), near-threshold auditory gap detection (Henry & Obleser, 2012), visual target detection (Kösem & Van Wassenhove, 2012; Mathewson, Fabiani, Gratton, Beck, & Lleras, 2010), and leads to faster reaction times in an oddball task in macaque monkeys (Lakatos et al., 2008).

Together, these studies show that temporal regularities can guide attention to optimize task performance. Considering the facilitating effects of perceived regularity, it has been proposed that the attentional system might prioritize structured information over more random sources (Yu & Zhao, 2015; Zhao, Al-Aidroos, & Turk-Browne, 2013). Indeed, Zhao et al. (2013) found that this was the case for order regularities. They showed that attention was biased towards the location and low-level visual features of shapes that appeared with a regular order, even when these regularities were not relevant for the task at hand. In Zhao et al.’s paradigm, participants performed search tasks in which they had to indicate the orientation of a T-shaped target presented among three L-shaped distractors. Crucially, the search tasks were interleaved by sequences of symbols. In three experiments, symbols with a certain feature (e.g., red) were presented in a fixed order, whereas symbols with a different feature (e.g., green) appeared in a random order. While the order regularity did not predict the location or timing of the target in the search task, Zhao et al. found faster reaction times when the features of the targets matched the regular symbols, indicating a bias towards these order regularities. They concluded that the implicit regularity in the task biased attention towards features associated with the regularity in a way that is not stimulus-identity driven, but also not driven by intentional goals.

While these results suggest that the attentional system is spontaneously tuned to order regularities, it is as of yet unknown whether this is also true for metrical temporal regularities—that is, for stimuli that occur isochronously. The goal of the current study is to investigate this question, by testing whether there is an attentional bias towards temporal regularities that are implicit (i.e., the participants are not informed about the existence of any regularities) and irrelevant for the task at hand. In six experiments based on the paradigm of Zhao et al. (2013), we tested whether attention was biased towards the location and color of the temporally regular-appearing stimuli. Participants were asked to perform search tasks in which a target appeared in one of four locations. In Experiment 1a, which was modeled after Experiment 1 of Zhao et al. (2013), sequences of squares were presented in between the search displays in the same four locations. Crucially, in one location, the square appeared with a regular rhythm. The temporal structure was uninformative about the visual search task in Experiment 1a. In contrast, in Experiment 1b, the structure of the regular stream could be used to predict target location in the search task. To test whether the speed and complexity of the regularity influence a potential attentional bias, both factors were manipulated in Experiment 1c and 1d.

Whereas location was the defining structural feature in Experiment 1, in Experiment 2, which was modeled after Zhao et al.’s (2013) Experiment 2, we investigated whether attention was spontaneously biased towards color features associated with temporal structure. A sequence of colored circles was presented in between the search trials, of which one specific colored circle appeared at regular intervals. Similar to Experiment 1c, we tested the influence of presentation speed in Experiment 2b. We expected that, if attention is automatically biased towards the temporal regularities, reaction times would be faster if the target matches the location (Experiment 1) and color (Experiment 2) of the regular stimulus.

## Experiment 1: Spatial bias

### Experiment 1a

#### Method

##### Participants

Forty-eight participants enrolled in the bachelor psychology program at the University of Groningen (24 female, *M*_age_ = 21.0 years, range: 18–29 years) participated in the experiment in exchange for course credits. The Psychology Ethical Committee of the University of Groningen approved the experimental protocol (16030-S-NE). All participants gave written informed consent prior to the experiment. The participants were naïve to the purpose of the study, but received a debriefing after the experiment.

#### Stimuli

##### Square stream

**Fig. 1:**
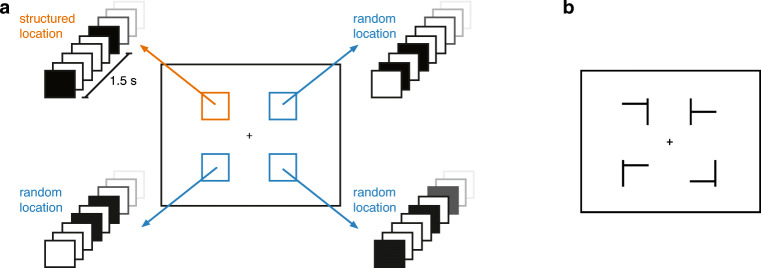
**a** Overview of the stimuli in Experiment 1a. Black squares appeared briefly at four locations on the screen. In one of the locations (counterbalanced over participants, but the top left in this example), the square appeared with a regular rhythm with an ISI of 1.5 s (i.e.**,** the structured stream). In the other three locations, the interval between the squares was random, but always 0 s, 0.3 s, 0.6 s, 0.9 s, or 1.2 s relative to the onset of the square in the structured location. **b** The presentation of squares was occasionally interleaved by a visual search display, in which the task was to find the target T shape among distractor L shapes, and indicate its orientation

The square stream consisted of black squares presented over four locations centered 5.1° from the central fixation cross: top-left, top-right, bottom-left, and bottom-right of the central fixation cross (see Fig. 1a). In one of the locations, the square was presented with a regular interonset time interval (the structured location), while the squares in the other three locations were presented with a random time interval (the random locations). All shapes had a size of 3.3° and were presented on a white background.

##### Visual search task

The visual search displays consisted of one T-shaped target and three L-shaped distractors (see Fig. 1b). All shapes had a size of 3.3°. The four shapes were presented in the same four locations as the square stream. The target T shape could point left (i.e., rotated 90°) or right (i.e., rotated 270°). The direction of the target was counterbalanced. The distractors consisted of an L shape or an inverted L shape, rotated 0° or 270°. The pointing direction of the distractors and target was counterbalanced in each visual search display, so that always two shapes pointed to the left and two shapes pointed to the right. The location of the shapes was randomized for each trial.

##### Apparatus

Stimuli were presented on a 1,280 × 1,024 Iiyama ProLite G2773HS screen with a refresh rate of 100 Hz. The experiment was built using Psychtoolbox-3 (Brainard, 1997; Kleiner, Brainard, & Pelli, 2007) in MATLAB 2015.

##### Procedure

At the start of the experiment, participants were instructed that they would complete search tasks by finding the target T shape among three distractor L shapes and indicating whether it pointed to the left or the right as quickly and accurately as possible. In addition, the participants were given the instruction that, between the search trials, they had to focus on the screen while task-irrelevant squares are presented.

In the structured location, the square was presented rhythmically with an interstimulus interval (ISI) of 1.5 s. In the other three, random locations, the same total number of squares was presented over the course of the experiment. However, the timing of the presentation of these squares was randomized, with the constraint that their onset could only be 0 s, 0.3 s, 0.6 s, 0.9 s, or 1.2 s relative to the onset of the square in the structured location. In total, a square was presented 1,120 times in each of the four locations. The structured and the random squares were always presented for 0.05 s.

Interrupting the square stream, 120 search trials were displayed over the course of the experiment. Each search trial consisted of a 0.75-s presentation of the visual search display, followed by a 0.75-s presentation of a central fixation cross. During the search trial, the participant could indicate whether the T shape pointed to the left or right by pressing the Z or the M key, respectively. If the participant did not give a response during the presentation of the search trial, the central fixation cross was presented until a response was given. The onset of the search trials was random, with the constraint that an equal number of trials was presented at five different onset intervals relative to the square in the structured stream: 0 s (i.e., the search trial appeared at the expected onset of the structured square), 0.3 s, 0.6 s, 0.9 s, or 1.2 s. Thus, over the course of the experiment, 24 search trials were presented at each of these potential onset intervals.

The experiment was divided into four equal-size blocks. In between the blocks, participants were instructed that they could take a break before continuing. After the experiment, participants filled in a short questionnaire in which they indicated whether they had noticed a pattern in the search trials or in the flashing black squares. For both questions, if they indicated that they had noticed a pattern, they were asked to describe the pattern. After this, participants were informed about the regular nature of the square in one of the four locations and asked to identify the structured location (the experiment script is available at https://osf.io/pnc4q/).

#### Results

##### Target location

**Fig. 2:**
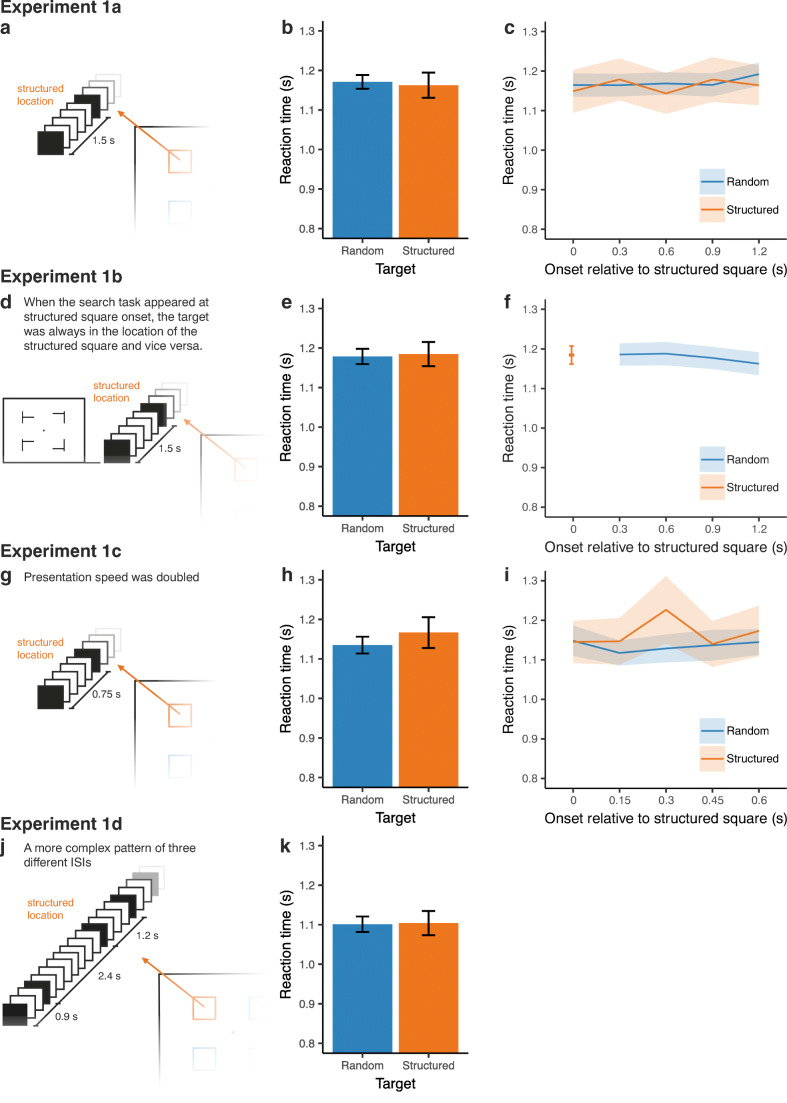
**a** Temporal regularity at the structured location in Experiment 1a. A black square was presented isochronously with an ISI of 1.5 s, whereas the same black square was presented with random timing at the random locations. **b** Average reaction times for targets in the structured or random locations in Experiment 1a. **c** Average reaction times for targets in the structured or random locations as a function of target onset, relative to the onset of the structured square (e.g., 0 indicates that the search task appeared at the moment that the square would otherwise appear at the structured location). **d** Change of the procedure in Experiment 1b, compared with Experiment 1a. **e** Average reaction times for targets in the structured or random locations in Experiment 1b. **f** Reaction times for the different onsets relative to the structured square in Experiment 1b. Given the more informative nature of the task, the target at the structured location always appeared at *t* = 0 (i.e.**,** the moment that the structured square would have appeared). **g** Change of the procedure in Experiment 1c, compared with Experiment 1a. **h** Average reaction times for targets in the structured or random locations in Experiment 1c. **i** Average reaction times for targets in the structured or random locations as a function of target onset in Experiment 1c. **j** Change of the procedure in Experiment 1d, compared with Experiment 1a. **k** Average reaction times for targets in the structured or random locations in Experiment 1d. In all figures, error bars represent within-subject confidence intervals (Morey, 2008)

Figure 2b shows the average reaction time for the structured location compared with the random location. To test whether participants were faster in the structured location than in the random location, we created linear mixed models (LMMs) using the lme4 package (Version 1.1-10; Bates, Mächler, Bolker, & Walker, 2015) in R Version 3.2.2 (R Development Core Team, 2008). In addition to the LMMs, in order to quantify the evidence in favor of the null hypothesis, we calculated Bayes factors using the lmBF function from the BayesFactor package in R (Morey, Rouder, & Jamil, 2014). We will denote the evidence for the null hypothesis (H_0_) over the alternative hypothesis (H_1_) as BF_01_. Only correct responses were included in the analysis. In addition, reaction times higher than 4 s were excluded from analysis. Reaction time was entered as the dependent variable, and subject was entered as a random factor. A variable coding whether the target was in the structured location was entered as a fixed factor. To control for a potential advantage of the actual location of the target, target location (top left, top right, bottom left, or bottom right) was also included as a fixed factor. In addition, the random slope of target location improved the model, and was therefore included (all analysis scripts and data are available at https://osf.io/pnc4q/).

We found no difference in reaction time when the target appeared in the structured location compared with the random locations (β = −0.02, *t* = −0.52, *p* = .601, BF_01_ = 21.09). Including target location improved the model significantly (*χ*^2^(3) = 43.76, *p* < .001, BF_01_ < 0.01). However, after inclusion of random slopes for target location, a post hoc Tukey’s HSD test showed that no difference in reaction time between the locations reached significance (*p*s > .068), suggesting no or only minor global effects of target location on reaction time.

##### Target onset

Figure 2c shows the reaction time for targets at the structured and the random location at the different onset times relative to the presentation of the rhythmic square. To test whether responses were faster at the moment of structured location onset, we compared an LMM including onset time as a fixed factor with a model excluding this factor. We found that the inclusion of onset time did not improve the fit of the model (*χ*^2^(4) = 2.63, *p* = .622, BF_01_ > 100).

##### Accuracy

Average accuracy for the orientation of the targets in the visual search task was 85.95% (*SD* = 9.69).

##### Questionnaire

Ten participants reported that they had noticed a pattern in the appearance of the squares, but none of the participants reported the rhythmic appearance of the square in one of the locations. When asked to identify the structured location after the experiment, 20.83% of the participants identified the correct location (chance level: 25%).

### Experiment 1b

In Experiment 1a, we investigated whether attention was biased towards a location in which a stimulus appeared with temporal regularity. The results of the search task showed that there was no reliable difference in reaction time between this structured location and locations containing no regular timing. These findings suggest that attention was not spontaneously biased towards the implicit regularity. As we intended to test the spontaneous attentional bias towards temporal regularity, the structure in Experiment 1a was not informative about the onset of the search task, nor of the location and orientation of the target. That is, participants could not use the structure to decrease their response times or predict where the target would appear. This leaves open the question whether participants would be biased towards the implicit regularity when it can be used to optimize performance in the search task. To this end, in Experiment 1b, we manipulated the predictability of the search task, so that trials in which the target appeared at the structured location exclusively appeared at the expected onset of the structured square. Thus, in this case, the location of the target could be predicted by the onset of the search task, increasing the utility of attending to the temporal regularity.

#### Method

Forty-four participants enrolled in the psychology bachelor program at the University of Groningen (23 female, *M*_age_ = 20.5 years, range: 17–26 years) participated in the experiment in exchange for course credits. Stimuli, apparatus, and procedure were similar to Experiment 1a. In contrast to Experiment 1a, however, the timing of the onset of the visual search task was predictive of the location of the target: When the search task appeared at structured square onset, the target was always in the location of the structured square and vice versa (see Fig. 2d). Thus, the 25% of the trials in which the target was at the structured location was presented at structured square onset, and the other 75% of the trials was presented 0.3 s, 0.6 s, 0.9 s, or 1.2 s after the onset of the structured square.

#### Results

##### Target location

Figure 2e shows the average reaction time for targets at the structured and random locations. The same LMM as in Experiment 1a was performed. We found no difference in reaction time for targets in the structured or the random location (β = 0.02, *t* = 0.65, *p* = .518, BF_01_ = 22.51). Again, including target location improved the model (*χ*^2^(3) = 46.86, *p* < .001, BF_01_ < 0.01). A post hoc Tukey’s HSD test showed faster reaction times for the two top locations compared with the bottom left location (*p*s < .046). No other contrasts reached significance (*p*s > .201).

##### Accuracy

Average accuracy in the search tasks was 84.24% (*SD* = 10.64).

##### Questionnaire

Seven participants reported that they had noticed a pattern in the presentation of the squares, but none of the participants reported that the square appeared rhythmically in one of the locations. In the forced-choice questionnaire, 20.45% of the participants identified the correct structured location (chance level: 25%).

### Experiment 1c

In Experiments 1a and 1b, the isochronous stimulus was presented with an ISI of 1.5 s. One reason for the absence of an attentional bias towards this stimulus in these experiments might be that the presentation rate was too slow to (implicitly or explicitly) notice the regularity: The integration of the statistical regularity of stimuli might become more difficult when they are presented with a long ISI. For example, attentional entrainment studies have employed presentation rates faster than the current 0.66 Hz (e.g., Henry & Obleser, 2012; Jones et al., 2002; Mathewson et al., 2010). To test this hypothesis in the current experiment, we increased the presentation rate of squares at the structured and the random location. To balance the increase of speed with the ability to present the random stimuli at time slots between the regular stimulus, we doubled the speed compared with Experiments 1a and 1b.

#### Method

Twenty-seven participants (21 female, *M*age = 23.8 years, range: 19–31 years) participated in the experiment in exchange for a 7-euro payment. Stimuli, apparatus, and procedure were similar to Experiment 1a. In the current experiment, however, the presentation speed of the square at the structured location was twice as fast as in Experiment 1a: the ISI was 0.75 s instead of 1.5 s (see Fig. 2g). To keep the duration of the experiment, and the average duration between two consecutive search trials, the same as in Experiment 1a, the number of presented squares at the structured and random location was doubled (i.e., a total of 2,240 presentations at each location).

#### Results

##### Target location

Figure 2h shows the average reaction time for the search task with the target appearing in the structured and the random location. The same LMM as in Experiments 1a and 1b showed that the reaction time did not differ between the structured location and the random locations (β = 0.02, *t* = 0.64, *p* = .526, BF_01_ = 3.99). Again, including target location improved the model (*χ*^2^(3) = 32.36, *p* < .001, BF_01_ < 0.01). However, no post hoc contrasts between the locations reached significance (*p*s > .147).

##### Target onset

Figure 2i shows the reaction time as a function of the timing of the search task relative to the onset of the structured square. We found that including onset time in the LMM did not improve the model fit (*χ*^2^(4) = 2.35, *p* = .672, BF_01_ > 100).

##### Accuracy

Average accuracy in the search tasks was 80.94% (*SD* = 14.62).

##### Questionnaire

Ten participants reported that they had noticed a pattern in the presentation of the squares, but none of the participants reported noticing the rhythmic appearance of the square in one location. In the forced-choice question, 18.52% of the participants identified the correct location (chance level: 25%).

### Experiment 1d

The regularity in Experiments 1a, 1b, and 1c consisted of a simple isochronous stimulus. This regularity could be considered as simpler than the order regularities of Zhao et al. (2013). In interacting with their environment, humans might optimize learning by attending to medium levels of complexity instead of stimuli that are either too predictable or too complex (the “Goldilocks effect”: Kidd, Piantadosi, & Aslin, 2012, 2014). In the current experiment, we therefore increased the temporal complexity of the rhythmic stimulus for closer correspondence with Zhao et al.’s work.

#### Method

Thirty-five participants (29 female, *M*_age_ = 19.57 years, range: 18–23 years) participated in the experiment in exchange for a 7-euro payment. Stimuli, apparatus, and procedure were similar to Experiment 1a. However, instead of an isochronous rhythm, the presentation of the square at the structured location followed a more complex repeating pattern. The following series of ISIs was repeated: 0.9 s, 2.4 s, 1.2 s (see Fig. 2j).

#### Results

##### Target location

Figure 2k shows the average reaction time for search trials in which the target was in the structured or random location. The same LMM as in the previous experiments showed that there was no evidence for a difference in reaction times between these locations (β = −0.01, *t* = −0.21, *p* = .838, BF_01_ = 22.36). Target location did not improve the model (*χ*^2^(3) = 6.29, *p* = .099, BF_01_ = 85.69).

##### Accuracy

The average accuracy in the search tasks was 78.92% (*SD* = 18.80).

##### Questionnaire

Eight participants reported noticing a pattern in the structured squares; however, no participants reported seeing a repeated rhythmic pattern in one particular location. When asked to identify the structured location, 17.14% of the participants were correct (chance level: 25%).

### Discussion Experiment 1

In Experiment 1, we tested whether attention is spontaneously biased towards temporal regularities. In four studies, we presented a stimulus with a temporal regularity in one location on the screen. We hypothesized that if attention was biased towards this location, reaction times to targets presented in this location would be faster. However, we found no evidence for a difference in reaction times between the structured and the random locations.

Three critical features of Experiment 1a might have prevented an attentional bias, which we subsequently manipulated in follow-up experiments. First, whereas attention was not spontaneously biased towards regularity, it might be biased when the temporal structure is useful for the task at hand. In Experiment 1b, we tested whether adding temporal predictability to the search task would increase a potential attentional bias towards the regular stream. However, the results again showed no decreased response time in the structured location, indicating that attention was not biased towards the structured stream. Thus, even when the temporal structure could be used to optimize task performance, no attentional bias was observed.

Second, previous experiments showing entrainment of attention to isochronous stimuli, have often used slightly faster presentation rates than the ISI of 1.5 s used in Experiments 1a and 1b (e.g., Henry & Obleser, 2012; Jones et al., 2002; Mathewson et al., 2010). In Experiment 1c we therefore doubled the presentation rate, to test whether this might induce an attentional bias. Yet, in line with Experiment 1a and 1b, we found no difference in reaction time between the structured and random location, nor did we find entrainment effects (i.e., the reaction time did not depend on the onset of the search task) and, as in Experiments 1a and 1b, participants were unable to report the nature of the regularity.

Third, given the proposed inverted U-shaped relation between stimulus complexity and attention (Kidd et al., 2012, 2014), the regularity in Experiment 1a might have been too simple to bias attention. In Experiment 1d, we therefore replaced the simple isochronous stimulus with a more complex pattern consisting of three consecutive ISIs. In line with the previous experiments, however, we found no evidence for spontaneous increased attention towards this pattern. Overall, these four experiments consistently showed that attention was not spontaneously biased towards temporal regularities and that participants were unable to explicitly report the nature of the temporal structure. In addition to the notion of regularity complexity, the use of simpler stimuli (squares) compared with the complex shapes in Zhao et al. (2013) could induce more peripheral processing, requiring less attention towards one particular spatial stream. Although also in Zhao et al. (2013) “participants were instructed to fixate while attending to the four locations” (p. 669), this potential difference in spatial attention may reduce a spatial bias. In Experiment 2, however, we will present colored stimuli in the center of the screen, so that these differences in spatial processing do not play a role.

While Zhao et al. (2013) found a spatial bias towards a stream containing regularly ordered stimuli, they also showed that this bias could be generalized to features other than location. For example, they showed that when a colored structured stream was interleaved with a random stream in a different color, there was a bias towards the structured color. Zhao et al. (2013) found that, in general, responses were faster when the target was colored compared with when a distractor was colored. Crucially, however, this difference was larger for the color associated with the structured compared with the random stream.

While we found no evidence for an attentional bias towards the location of the regular stimulus in Experiment 1, it is possible that a bias exists towards other features associated with temporal regularity. Therefore, in Experiment 2, we tested whether attention is biased towards the color of a temporally structured compared to a random stream. A stream of circles was presented in the center of the screen. The circles appeared in two colors: orange and blue. One of these colors appeared always after a regular interonset interval, while the other color appeared equally often, but with a random timing. Occasional visual search tasks appeared in which one of the four items was colored. We expected faster reaction times when the target stands out by color compared with when a distractor is colored. However, if attention is biased towards the features of the regular stream, we expected that this difference in reaction times is larger for the structured color compared with the random color.

## Experiment 2: Color bias

### Experiment 2a

#### Method

##### Participants

Forty-five participants enrolled in the psychology bachelor program at the University of Groningen (37 female, *M*_age_ = 20 years, range: 17–26 years) participated in the experiment in exchange for course credits. The Psychology Ethical Committee of the University of Groningen approved the experimental protocol (16030-S-NE). All participants gave written informed consent prior to the experiment. The participants were naïve to the purpose of the study, but received a debriefing after the experiment. Two participants were excluded from analysis, because they did not adhere to task instructions.

#### Stimuli

##### Circle stream

**Fig. 3:**
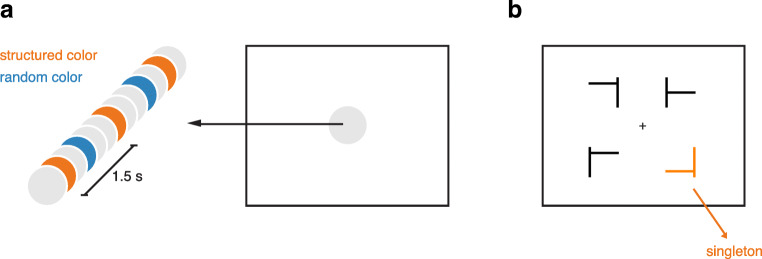
**a** Overview of the stimuli in Experiment 2a. A sequence of circles was presented at the center of the screen. One colored circle appeared with a fixed rhythm (counterbalanced over participants, but the orange circle in this example), whereas the other colored circle appeared equally often, but with random timing (here, the blue circle). **b** Occasional search displays appeared, in which one of the shapes was colored either blue or orange: the singleton. We refer to the singleton as structured if it matched the structured color, and random if it matched the random color. The singleton could be the target T shape or one of the distractor L shapes. (Color figure online)

The circle stream consisted of a sequential presentation of an orange and blue circle at the center of the screen (see Fig. 3a). The size of the circles was 3.3°, and the orange color was luminance-matched to the blue color (RGB values: 0, 127, 255) using a luminance meter. One of the colored circles was presented with a regular interonset time interval (the structured color), while the other colored circle was presented with a random time interval (the random color). The structured color (i.e., either orange or blue) was counterbalanced over participants. In between the presentations of the structured and random colored circles, a light-gray circle was presented as a fixation stimulus (RGB values: 230, 230, 230). All stimuli were presented on a white background.

##### Visual search task

Visual search displays were identical to Experiment 1a and 1b. However, whereas three shapes in the visual search displays were presented in black, one of the shapes was colored orange or blue: the singleton (see Fig. 3b). In 50% of the trials, the singleton was the same color as the structured circle, and in the other 50% of the trials, the singleton was the color of the random circle. For both singleton colors, the singleton was the target in 25%, and the distractor in 75% of the trials. Thus, the singleton was not informative about the target location or orientation.

##### Apparatus

Apparatus was similar to Experiments 1a-d.

##### Procedure

At the start of the experiment, participants were instructed that they would complete search tasks, by finding the target T shape among three distractor L shapes and indicating whether it pointed to the left or the right as quickly and accurately as possible. They were instructed that the color of the singleton did not predict the target location. In addition, the participants were given the instruction that between the search trials they had to focus on the task-irrelevant circles at the center of the screen.

The structured circle was presented rhythmically with an ISI of 1.5 s. The timing of the presentation of the random circle was random, but always 0.3 s, 0.6 s, 0.9 s, or 1.2 s after the onset of the structured circle. The structured and the random circle were always presented for 0.15 s. In total, both circles were presented 1,120 times. In between the presentations of the structured and random circle, the gray circle was presented.

Interleaving the circle stream, 160 search trials were displayed over the course of the experiment. Each search trial consisted of a 0.75 s presentation of the visual search display, followed by a 0.75 s presentation of a central fixation cross. During the search trial, the participant could indicate whether the T shape pointed to the left or right by pressing the Z or the M key, respectively. If the participant did not give a response during the presentation of the search trial, the central fixation cross was presented until a response was given. The onset of the search trials was random, with the constraint that an equal number of trials was presented at five different onset intervals relative to the structured circle: structured circle onset, 0.3 s, 0.6 s, 0.9 s, or 1.2 s after structured circle onset. Thus, over the course of the experiment, 32 search trials were presented at each of these potential onset intervals.

The experiment was divided into four equal size blocks. In between the blocks, participants were instructed that they could take a break before continuing. After the experiment, participants filled in a short questionnaire in which they indicated whether they had noticed a pattern in the search trials or in the colored circles. If they had noticed a pattern, they were asked describe it.

#### Results

##### Target color

**Fig. 4:**
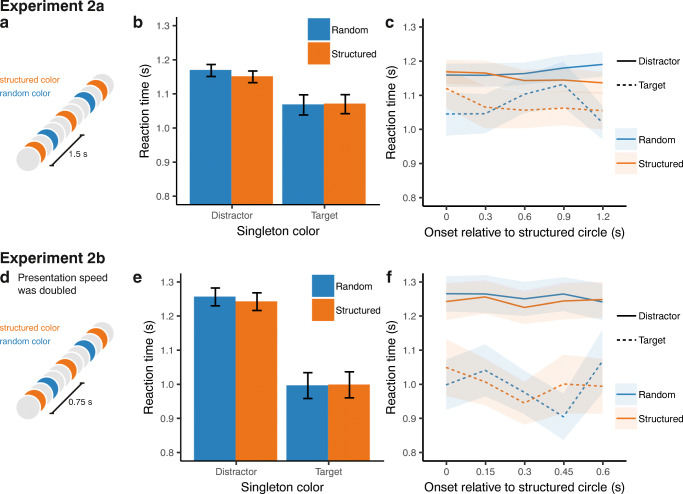
**a** Overview of the stimuli presented at the center of the screen in Experiment 2a. **b** Average reaction times for the search task in Experiment 2a, in which the colored singleton could be either the target or a distractor. In addition, the singleton could be presented in the structured or the random color. **c** Average reaction times as a function of the onset relative to the presentation of the circle with the structured color in Experiment 2a. **d** Stimuli presented at the center of the screen in Experiment 2b. The presentation speed was twice as fast as in Experiment 2a. **e** Average reaction times in Experiment 2b for the singleton targets and distractors, presented in the random or structured color. **f** Average reaction times as a function of the onset relative to the presentation of the circle with the structured color in Experiment 2b. In all figures, error bars represent within-subject confidence intervals

Figure 4b shows the average reaction time for whether the singleton was the structured or random color and whether the singleton was the target or one of the distractors. An LMM was performed with reaction time as the dependent variable and subject as a random factor. Singleton color (structured or random) and singleton type (distractor or target) were entered as fixed factors. To control for a potential advantage of the actual location of the target, target location (top left, top right, bottom left, or bottom right) was also included as a fixed factor. The random slope term for target location improved the model fit and was included in the final model. Only correct responses were included in the analysis. In addition, reaction times higher than 4 s were excluded from analysis.

When the colored singleton was the target, participants responded faster than when the singleton was a distractor (β = −0.10, *t* = −6.83, *p* < .001, BF_01_ < 0.01). However, there was no difference between the reaction times between the structured and random color (β = −0.01, *t* = −1.36, *p* = .174, BF_01_ = 24.56). We expected that attention would be spontaneously biased towards the color of the structured circle, which would hypothetically lead to a faster reaction time for the structured color target and a slower reaction time for the structured color distractor (i.e., an interaction effect of singleton color and singleton type). However, this predicted interaction was not observed (β = 0.02, *t* = 0.85, *p* = .398, BF_01_ = 13.85).

##### Target location

Again, including target location improved the model significantly (*χ*^2^(3) = 42.88, *p* < .001, BF_01_ < 0.01). A post hoc Tukey’s HSD test showed that reaction times to targets appearing at the top right position were faster than at the bottom right position (*p* = .046). No other contrasts reached significance (*p*s > .060).

##### Target onset

Figure 4c shows the average reaction time in the different singleton conditions for the five possible onsets relative to the structured color circle. Including target onset, relative to the structured circle, did not improve the fit of the model (*χ*^2^(4) = 2.04, *p* = .728, BF_01_ > 100).

##### Accuracy

Average accuracy in the visual target detection task was 87.76% (*SD* = 8.62).

##### Questionnaire

Nine participants reported that they had noticed a pattern, but none correctly reported the rhythmic nature of the structured circle.

### Experiment 2b

In a similar manipulation as in Experiment 1c, we doubled the presentation rate of the stimuli in Experiment 2b. This resulted in an ISI of 0.75 s between two consecutive structured color stimuli.

#### Method

Twenty participants enrolled in the psychology bachelor program at the University of Groningen (11 female, *M*_age_ = 23.2 years, range: 18–30 years) participated in the experiment in exchange for course credits. The procedure was similar to Experiment 2a; however, in a manipulation similar to Experiment 1c, we doubled the presentation speed (i.e., an ISI 0.75 s for the rhythmic color) and the number of presented circles. Thus, the total duration of the experiment, as well as the average interval between consecutive search trials, was identical to Experiment 2a.

#### Results

##### Target color

Figure 4e shows the average reaction times for the different singleton conditions. The same LMM as in Experiment 2a was performed. The model showed faster reaction times for singleton targets compared to singleton distractors (β = −0.25, *t* = −11.96, *p* < .001, BF_01_ < 0.01). There was no difference in reaction time between the structured and the random color (β = −0.01, *t* = −0.94, *p* = .346, BF_01_ = 18.24). In line with Experiment 2a, the effect of singleton type was not stronger for the structured compared to the random color (β = 0.01, *t* = 0.42, *p* = .676, BF_01_ = 12.08).

##### Target location

Including target location did not improve the model significantly (*χ*^2^(3) = 2.71, *p* = .438, BF_01_ > 100), indicating that the reaction time did not differ between the four locations in which the target could appear.

##### Target onset

Figure 4f shows the reaction time for the different search trial onsets relative to the structured circle. Model comparison showed that the response time did not depend on the onset of the search task relative to the structured circle (*χ*^2^(4) = 3.56, *p* = .470, BF_01_ > 100).

##### Accuracy

Average accuracy in the search tasks was 87.43% (*SD* = 14.09).

##### Questionnaire

Six participants reported a to have noticed a pattern in the colored circles, but none identified the rhythmic nature of one particular color correctly.

### Discussion Experiment 2

In Experiment 2a, we have tested whether attention was spontaneously biased towards the color features associated with an isochronous stimulus. As expected, we found that reaction times were faster when the colored singleton was the target than when it was a distractor. However, this difference was similar for the structured color and the random color. In addition, overall reaction times were similar for the structured compared to the random color. Decreasing the ISI of the isochronous, regular stimulus (in Experiment 2b) did not affect this pattern of results. In line with Experiment 1, these findings do not provide evidence that participants were biased towards the features associated with temporal structure.

## General discussion

We aimed to test whether attention is biased towards implicit metrical temporal regularities. In that case, we expected faster reaction times when targets in the search tasks matched the features of the regular stimulus. However, we found no difference in reaction time between the structured and random location (Experiment 1) or color (Experiment 2), failing to support the hypothesis that the present temporal regularity is prioritized over the random streams. We instead found some evidence that reaction times were faster for targets presented at the top of the search screen compared with the bottom. In addition, response time did not depend on the onset of the search task relative to the structured stream, suggesting that attention was not entrained to the isochronous stimulus.

Overall, our results show that attention was not biased towards the temporal regularities in the task. Thus, whereas previous studies have shown that temporal structure can be used to optimize attention and perception when the regularities reliably predict upcoming stimuli (Correa, Lupiáñez, & Tudela, 2005; ; Martens & Johnson, 2005; Niemi & Näätänen, 1981; Olson & Chun, 2001; Willems, Damsma, Wierda, Taatgen, & Martens, 2015), the current results suggest that this might not be the case when such regularities are uninformative about the task at hand. A potential explanation is that, in the latter case, prioritizing attention to temporal regularities might actually be detrimental for task performance. Indeed, Schroeder and Lakatos (2009) proposed that the brain can operate in either a “rhythmic” or a “continuous” mode, depending on the nature of the task. The rhythmic mode is activated when the task contains a task-relevant rhythm, resulting in low-frequency entrainment of the sensory cortex and, thereby enhanced perceptual sensitivity to stimuli that are in phase with the rhythm. However, if the task contains no relevant rhythm, the brain can operate in continuous mode. By suppressing low-frequency oscillations and enhancing gamma-band oscillations, a more continuous state of vigilance is achieved to deal with the temporal unpredictability of the upcoming stimuli. In this way, the cost of lower sensitivity in the low-excitability phase of the neural entrainment can be prevented.

A similar argument can be made to explain the apparent discrepancy between our results and Zhao et al. (2013), who found that attention was spontaneously biased towards features of a stream with a regular order. They proposed that attention and statistical learning could act in a closed-loop way: The fulfillment of predictions based on previous learning might increase attention, which in turn enhances learning. Crucially, however, we have shown here that this bias does not generalize to metrical temporal regularities. Although the statistical learning of order regularities might bias attention towards associated features, it does not necessarily interfere with the processing of the unpredictable search task. An attentional bias towards a rhythmic visual stimulus, in contrast, may come at the cost of diminished continuous sensitivity. In Experiment 1b, we tested whether there was a bias towards regularities when they were partly informative about the onset of the search task. In this experiment, trials in which the target appeared at the structured location always appeared at the expected onset of the structured square. While we found that there was still no attentional bias in this case, the manipulation only added predictability for part of the trials (i.e., trials that appeared at one particular phase), and attentional entrainment might therefore not have contributed significantly in optimizing task performance.

Although neural oscillations may reduce continuous sensitivity, previous studies have shown that attention can be guided by the phase of rhythmic stimuli, even when they are not necessarily related to the task at hand. For example, rhythmic stimuli have been shown to facilitate auditory and visual detection at specific phases in the rhythm (Bolger, Coull, & Schön, 2014; Henry & Obleser, 2012; Jones et al., 2002; Kösem & Van Wassenhove, 2012; Mathewson et al., 2010). Our findings suggest that these temporal phase biases do not generalize to nontemporal features of the regular stimuli, such as, in this case, location and color. However, we also did not find an effect of phase on reaction time. One potential explanation for this absence, as well as the general absence of an attentional bias, might be that the temporal regularities in our experiments were too implicit and could therefore not be learned. Indeed, in contrast to the entrainment studies, the present rhythmic stimulus was embedded in other stimulus streams, without explicit instruction to pay attention to the rhythmic stream. Our questionnaire data showed that participants did not explicitly learn the regularities, but the results do not provide conclusive evidence about the precise nature of the learning. As implicit learning may be a prerequisite for an attentional bias, future studies could assess implicit learning after the experiment with, for example, a two-alternative forced-choice task, in which participants have to pick the most familiar stimulus from a regular stimulus (as presented in the experiment) and a foil stimulus (Zhao et al., 2013).

A related point of consideration is the frequency at which the regular stimulus is presented. Whereas we used an ISI of 1.5 s (0.67 Hz, similar to the 0.5 Hz entrainment used by Bolger, Coull, & Schön, 2014) in Experiments 1a, 1b, 1d, and 2a, previous studies showing an entrainment effect have often used slightly higher frequencies (e.g., 3 Hz in Henry & Obleser, 2012; 1.67 Hz in Jones et al., 2002; 12 Hz in Mathewson et al., 2010). It is possible that the relatively low frequency impeded the detection of, or the entrainment to, the structured stimulus. Therefore, we also tested an ISI of 0.75 s (1.33 Hz, matching the range used by Kösem & Van Wassenhove, 2012, who showed improvements in the 0.6–1.4 Hz range) in Experiment 1c and Experiment 2b. The results, however, were similar to those of the slower paradigms, indicating that increasing the presentation rate did not enhance a potential attentional bias.

At first sight, the regularities presented in our experiments might seem less complex than the order regularities in Zhao et al. (2013). Zhao et al.’s stimuli consisted of three sets of three shapes that always appeared in the same order, contrasted by a randomly sequenced set of nine shapes. In contrast, our temporal regularities consisted of a simple isochronous stimulus. The complexity of a stimulus has been suggested to influence the attention and learning strategy of an observer, given the observer’s current mental representations (Berlyne, 1960; Dember & Earl, 1957). An optimal learning strategy in our dynamic world might involve seeking to minimize the prediction error and to maximize mutual information between the observers’ mental representations and the environment, leading to a preference for conditions that are neither too predictable nor too complex (Clark, 2017; Little & Sommer, 2013). At this “sweet spot” of optimal learning, cognitive resources are not wasted on stimuli that do not allow us to improve our understanding, and our predictions, of the world (Kidd & Hayden, 2015). Indeed, infants have been shown to pay most attention to visual event sequences of medium-level complexity, a phenomenon that has been dubbed the “Goldilocks effect” (Kidd et al., 2012, 2014).

Given the Goldilocks effect, the regularity in the current study could be too simple. This might have influenced a potential attentional bias in three ways. First, if we increase the complexity of the isochronous regularity, we would potentially find a spontaneous attentional bias. We tested this notion in Experiment 1d, in which the simple isochronous stimulus at the regular location was replaced by a square that was presented in a rhythmic, yet more complex pattern with three different, sequential ISIs. However, in line with Experiment 1a, the results showed no difference in reaction time between the structured and the random location. In addition, the questionnaire results showed that, also with this more complex stimulus, participants did not notice the regularity. Second, if the regularities were indeed simple, participants might have become aware of them, and this might have led to explicit attentional strategies. However, none of the participants in the six experiments reported the rhythmic nature of one of the stimuli in post-experiment questionnaires, indicating that participants were unaware of the temporal regularities. This is in line with Zhao et al. (2013), who found indications of explicit awareness of the order of regularities for only three out of 47 participants in Experiment 1 and 2. In addition, their response-time effects were still reliable after the exclusion of participants who had noticed any regularity. Third, the high predictability of the simple isochronous stimulus might have led to a decrease in attentional bias over time. Indeed, prolonged presentations of regularities might lead to habituation and, thereby, a decrease in attention to repeated stimuli (e.g., Turk-Browne, Scholl, & Chun, 2008). In this case, we might expect that the regularity initially attracts attention, but over time, habituation decreases attention to the predictable stimulus. Therefore, as a post hoc analysis, we tested the effect of habituation by adding experimental block (i.e., the experiments were divided in four blocks) to the mixed models. Whereas the models showed that the overall reaction time decreased over blocks (*χ*^2^s > 75.83, *p*s < .001, BFs_01_ < 0.01), we found that the difference in reaction time between the structured and random location did not decrease in Experiments 1a–1d (*χ*^2^s < 3.39, *p*s > .066, BFs_01_ > 3.93). In Experiments 2a and 2b, we found that the interaction between singleton type and color did not change over blocks (*χ*^2^s < 5.96, *p*s > .114, BFs_01_ > 100). Thus, we found no evidence for an initial attentional bias that decreased over the course of the experiment. Overall, these results suggest that the current regularities were not too simple with regard to the Goldilocks rule.

On the other hand, certain sequence and stimulus features may have hampered participants’ ability to pick up on the regularities. First, the similarity of the presented squares (in Experiment 1) and colored circles (in Experiment 2) might cause an automatic overwriting of items currently held in working memory (Alvarez & Thompson, 2009). Second, in Experiment 1, the presented squares at four locations on the screen were marked by abrupt visual onset. As it has been shown that object onsets capture attention (Yantis & Jonides, 1984) and may lead to automatically storing an object in working memory (Schmidt, Vogel, Woodman, & Luck, 2002), the onsets of the squares at the random location might have prevented an attentional bias towards the structured location. In this case, we would expect faster reaction times when, in one particular location, a target appears right after the presentation of a square. However, we did not find such a decreased response time when analyzing the effect of target onset relative to the structured square (in Experiments 1a and 1c). Future studies might investigate whether the detection of temporal regularities is improved by using sufficiently different stimuli, presented without a sharp onset.

In the current study, we have focused on rhythmic visual stimuli. However, compared with, for example, order or spatial regularities, a potential bias towards structured temporal information might be more apparent in the auditory than in the visual modality. Although there is abundant evidence for sensitivity to visual temporal regularities, rhythmic processing of auditory information has been shown to be more precise than, and dominant over, visual information (e.g., Chen, Repp, & Patel, 2002; Kolers & Brewster, 1985; Recanzone, 2003; Repp & Penel, 2002). Future studies could test whether these modality differences influence a potential attentional bias towards metrical temporal structure.

In summary, in six experiments we found strong evidence that attention was not spontaneously biased towards implicit temporal regularities when they were not relevant for the task at hand. Whereas people might optimize task performance by exploiting regularities, the processing of irrelevant features of temporally regular events does not seem to be prioritized.
